# The mechanism of dishabituation

**DOI:** 10.3389/fnint.2014.00014

**Published:** 2014-02-14

**Authors:** Genevieve Z. Steiner, Robert J. Barry

**Affiliations:** Centre for Psychophysics, Psychophysiology, and Psychopharmacology, Brain & Behaviour Research Institute, School of Psychology, University of WollongongWollongong, NSW, Australia

**Keywords:** dishabituation, orienting reflex, counting, electrodermal activity, habituation, sensitization

## Abstract

The dual-process theory of habituation attributes dishabituation, an increase in responding to a habituated stimulus after an interpolated deviant, to sensitization, a change in arousal. Our previous investigations into elicitation and habituation of the electrodermal orienting reflex (OR) showed that dishabituation is independent of sensitization for indifferent stimuli, arguing against dual-process theory’s explanation. However, this could not be tested for significant stimuli in that study, because sensitization was confounded with incomplete resolution of the preceding OR. This study aimed to clarify the mechanism of dishabituation for significant stimuli by extending the stimulus onset asynchrony (SOA) beyond the time required for the phasic response to resolve. Participants completed an auditory dishabituation task with a random SOA of 13–15 s while their electrodermal activity was recorded. The stimulus sequence was 10 standards, 1 deviant, 2–4 standards; counterbalanced innocuous tones. Two counterbalanced conditions were used: silently count all stimuli (significant) and no task (indifferent). Skin conductance responses (SCRs) and pre-stimulus skin conductance levels (SCLs) both decremented over trials 1–10. In both conditions, SCRs showed response recovery and dishabituation, indicating habituation, and post-deviant SCL sensitization was apparent. Across all trials, phasic ORs were dependent on the pre-stimulus SCL (arousal level); this did not differ with condition. Importantly, dishabituation was independent of sensitization for both conditions. Findings indicate that sensitization, the *change* in state, is a process separate from phasic response resolution, and that arousal consistently predicts OR magnitude, *including* the dishabituation response. This argues against dual-process theory’s explanation, and instead suggests that dishabituation is a disruption of the habituation process, with magnitude determined by the current arousal level.

## INTRODUCTION

The process of habituation, a simple form of learning where responding to a repetitious stimulus decreases, has been documented in a wide range of organisms (from single-celled animals to primates) and is thought to allow an organism to reflexively filter out irrelevant information. Habituation is unique from other decrementing processes such as fatigue or refractory periods, as it can be interrupted by a change in stimulation (Thompson et al., 1979). For a decrementing response pattern to be correctly classified as habituation, Thompson and Spencer (1966), and more recently, Rankin et al. (2009), suggest that *response recovery*, an enhanced response to a change-stimulus (deviant), and *dishabituation*, a post-deviant increase in response to the habituated stimulus, should be evident. This paper will focus on clarifying the mechanism underpinning the latter of these criteria, dishabituation, in the context of the orienting reflex (OR) captured by the human electrodermal response.

Over the past century, there have been several theories on elicitation and habituation of the OR, the adaptive mechanism responsible for directing an organism’s attention toward environmental changes (Barry, 2009). Arguably, the two most prominent of these theories are Sokolov’s (1960, 1963a,b) neuronal-model comparator theory, and Groves and Thompson’s (1970) dual-process theory. Sokolov theorized that an OR was elicited when an incoming stimulus did not match a neuronal representation of the environment, and this was extensively explored with habituation of the phasic electrodermal response (galvanic skin response, now the skin conductance response; SCR). Sokolov’s model also incorporated a response amplifier, where the arousal state of the organism, measured by the slow-changing skin conductance level (SCL; Barry and Sokolov, 1993), determined the phasic response. Dual-process theory outlines two hypothetical processes that reflect the novelty of a stimulus: habituation (H), a pathway-specific decremental process; and sensitization (S), a state process that increases to novel stimuli. These two processes interact to determine OR magnitude, with the S-process regulating the outcomes of the H-process and serving as a response amplifier.

In addition to documenting decreases in OR magnitude, Sokolov (1963b) also noted that ORs increased to task-relevant “*significant*” stimuli, relative to task-irrelevant “*indifferent*” stimuli, and habituated more slowly in the context of heightened arousal. Although several studies have confirmed this finding (Barry, 2004, 2009; Steiner and Barry, 2011), neither Sokolov’s neuronal model nor dual-process theory offer a theoretical mechanism to generate the arousal increases associated with stimulus significance. Alternatively, Maltzman’s (1979a,b, 1990) voluntary OR concept can predict the increased arousal and phasic responding related to significant stimuli by providing a mechanism of cortical activation. Maltzman argued that individuals generate a “cortical set” related to a range of factors including task instructions, prior learning, etc., and the voluntary OR is the outcome of the cortical activation associated with the individual’s cortical set. The current study aimed to test aspects of both Sokolovian and dual process theories in the context of indifferent and significant stimuli.

Both the neuronal-model comparator theory (Sokolov, 1963b) and dual-process theory (Groves and Thompson, 1970; outlined more recently in Thompson, 2009) make different predictions about the mechanism of dishabituation, with Sokolov suggesting that dishabituation is a disturbance in the habituation process, and dual-process theory predicting that dishabituation is a superimposed process of sensitization (an increase in state level/arousal). In our previous investigation into the mechanism of dishabituation (Steiner and Barry, 2011), we tested dual-process theory’s unique prediction: that dishabituation reflects nothing more than the sensitization process, a* change* in arousal. We found that dishabituation was independent of sensitization for indifferent stimuli, a finding that argues against dual-process theory’s mechanism. In that study, however, the same conclusion could not be drawn for significant stimuli: the phasic response to the deviant had not resolved before the dishabituation trial, and subsequently interfered with measurement of the sensitization process. The aim of the current study was to continue along the same line of investigation used in Steiner and Barry (2011) by extending the stimulus onset asynchrony (SOA) long enough to allow resolution of the deviant response preceding the dishabituation trial.

Following Steiner and Barry (2011), we made a similar set of predictions. We hypothesized that SCR would demonstrate habituation: decrement with stimulus repetition, response recovery to an interpolated deviant, and dishabituation to the re-presentation of the habituated stimulus, regardless of stimulus significance. It was also predicted that for significant stimuli, SCRs would be enhanced and decrement more slowly than for indifferent stimuli.

Pre-stimulus SCLs (arousal level) were also expected to decrement with stimulus repetition and be enhanced for significant stimuli. We expected sensitization, apparent as an increase in arousal, to follow the deviant stimulus and that this process would be independent of the deviant response for *both* indifferent and significant stimuli. Again following Steiner and Barry (2011), it was hypothesized that dishabituation would be independent of sensitization, this time for both conditions, a prediction which, if shown to be true, would argue against the dual-process theory’s mechanism of dishabituation. Also, and in line with Sokolov’s assertion that the current arousal level amplifies the phasic response, it was predicted that SCR would be dependent on the pre-stimulus SCL.

## MATERIALS AND METHOD

### PARTICIPANTS

Twenty-four undergraduate students participated in this study in return for course credit (age: 18–25 years, 23 right-handed, 14 males). All provided informed consent prior to participating, and were free to withdraw at any time without penalty. Participants self-reported no use of psychotropic medication, and no neurological or psychiatric illnesses. Self-reports also indicated that participants had refrained from psychoactive substances for at least 12 h and from tea, coffee, alcohol, and cigarettes for at least 2 h prior to testing. All participants had normal or corrected-to-normal vision and self-reported normal hearing.

### PROCEDURE

Participants completed a demographic and screening questionnaire, were fitted with electrodermal recording apparatus, seated in an air-conditioned room 600–800 mm in front of a 19″ Dell LCD monitor (REV A00) and instructed to fixate on a 10 mm × 10 mm gray cross displayed in the center of a black background. Acoustic stimuli were delivered binaurally through Sony MDR V700 circumaural stereo headphones, and consisted of 1000 and 1500 Hz tones, each of 50 ms duration (15 ms rise/fall time), 60 dB SPL, with a random SOA of 13–15 s. The stimulus sequence included 10 tones of one frequency (standards), a *deviant* tone of a different frequency, followed by 2–4 standards; the standard/deviant frequencies were counterbalanced between subjects. All participants completed two counterbalanced conditions presented approximately 3 min apart: Indifferent, where participants were instructed that there was “no task in relation to the sounds”; and Significant, where participants were directed to “silently count the sounds and report to the researcher at the end of the experiment”. This procedure was approved by the joint South Eastern Sydney/Illawarra Area Health Service and University of Wollongong Health and Medical Human Research Ethics Committee.

### MATERIALS AND APPARATUS

Electrodermal data were recorded from the distal volar surface of digits II and III of the non-dominant hand using sintered silver/silver-chloride (Ag/AgCl) electrodes, filled with isotonic electrode paste of 0.05 M NaCl in an inert ointment base. Skin conductance was sampled using a constant voltage device (UFI Bioderm model 2701) at 0.5 V. The DC-coupled skin conductance output was sampled at 1000 Hz using a Neuroscan Synamps 2 digital signal-processing system and Neuroscan 4.3.1 Acquire software, and stored on a Dell Optiplex 755 computer. Stimulus presentation was controlled by a similar linked stimulus computer using Neurobehavioral Systems Inc. Presentation V 13.0 Build 01.23.09 software.

### DATA EXTRACTION

**FIGURE 1:**
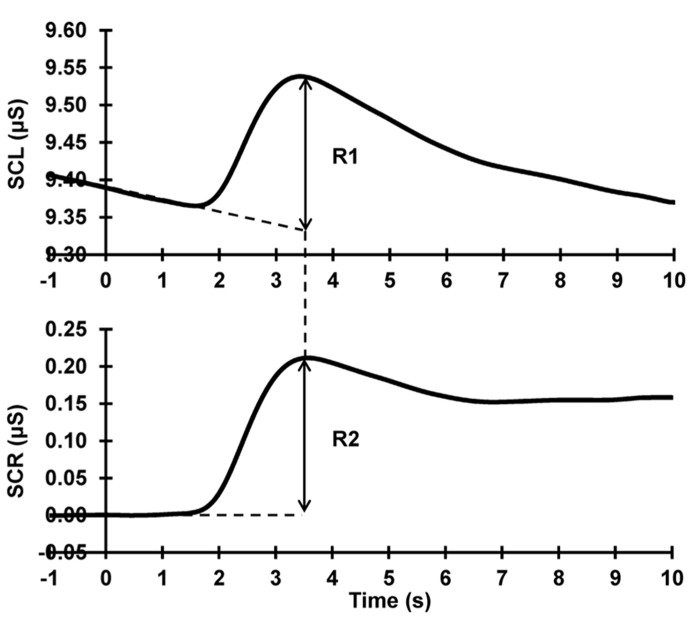
**SCR quantification procedure.** Top: raw data; Bottom: after baseline-de-trending. The dashed lines at the base of the response peak represent the falling and corrected baselines, respectively. The arrows illustrate the SCR peak amplitude quantification in each. It can be seen that R1 = R2, in terms of both amplitude and peak latency.

Raw data were band-pass filtered (0.1–3 Hz, zero-phase shift, 24 dB/Octave) and epoched offline 1 s pre- to 13 s post-stimulus using Neuroscan 4.3.1 Edit Software. An average of 1 s of immediately pre-stimulus activity was taken as a measure of SCL. Using the linear detrend function in Neuroscan on each trial, pre-response levels (1 s pre- to 1 s post-stimulus onset) were linearly extrapolated to compensate for falling baselines, following Barry et al. (1993). Each phasic response (with onset latency 1–3 s post-stimulus onset, following Barry, 1990) was quantified for each subject and each trial, as the difference between the extrapolated baseline and the maximum value of the subsequent peak (see **Figure 1**). SCRs were square-root transformed to reduce skew (Barry and Sokolov, 1993; Barry, 2004). Trials that contained outliers (such as non-stimulus related responses) were removed and replaced by an average of the trials preceding and succeeding the outlier trial.

### STATISTICAL ANALYSES

The design was a within-subject study of response decrement, response recovery, dishabituation, and sensitization for each condition. MANOVAs assessed the effect of condition across all 13 trials, separately for SCRs and SCLs. Separate MANOVAs were used to examine response decrement over trials 1–10 for both SCR and SCL with factors of condition (Indifferent vs. Significant) and trials (1–10); within trials, linear and quadratic trends were assessed. For SCR only, separate MANOVAs were carried out to assess response recovery and dishabituation, again with factors of condition (Indifferent vs. Significant) and trials (response recovery: 10 and 11; dishabituation: 10 and 12). MANOVA examined pre-stimulus SCL deviant sensitization with a comparison of trial 12–11 for both conditions. All *F*-tests are reported with (1, 23) degrees of freedom unless otherwise specified.

No Bonferroni-type α adjustment was required as contrasts were planned, and the number of contrasts did not exceed the degrees of freedom for effect (Tabachnick and Fidell, 1989). The violations of sphericity assumptions often associated with repeated-measures analyses do not affect single degree of freedom contrasts, so Greenhouse–Geisser-type correction was not necessary (O’Brien and Kaiser, 1985).

To test whether the post-deviant increase in SCL (deviant sensitization) was true sensitization (i.e., was not related to the incomplete resolution of the deviant response), a bivariate correlation compared the change in SCL (trial 12 minus 11) to the SCR at trial 11. To test dual-process theory’s unique explanation of dishabituation, a further correlation was calculated that compared the extent of dishabituation in the phasic OR (SCR trial 12 minus 10) to the sensitized arousal from the deviant stimulus (to ensure a consistent comparison with SCR, SCL was also calculated as trial 12 minus 10). To test Sokolov’s assertion that arousal is a response amplifier, SCRs were correlated with pre-stimulus SCLs across trials and subjects. To see how this changed over trials, separate correlations were also carried out for each trial. A multiple regression was also conducted to examine the OR determinants outlined by Sokolov, dual-process theory, and Maltzman (i.e., novelty, significance, and arousal) as predictors of SCR. As SCR was expected to reduce with novelty over trials, novelty was entered into the multiple regression as the reciprocal of each trial number (e.g., trial 10 was entered as 1/10). One-way tests were utilized for all analyzed predictions.

## RESULTS

Data from all participants were included as all reported the correct number of stimuli for the significant condition at the end of the condition.

### SKIN CONDUCTANCE RESPONSES

**FIGURE 2:**
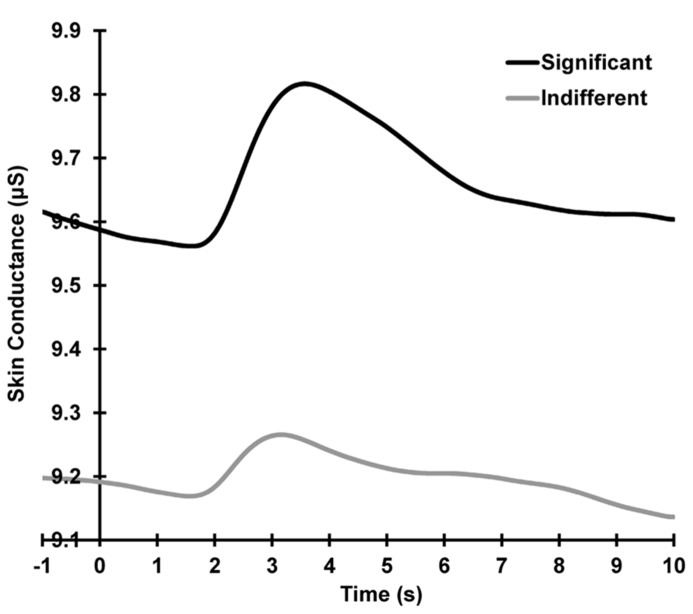
**Grand mean SCRs across all trials, separately for each condition.** Activity shown is prior to linear de-trending to remove the falling baselines.

**Figure 2** shows the grand mean raw SCR waveforms across all subjects and trials, separately for each condition. A condition main effect is apparent, with larger mean baselined SCRs for the significant compared to the indifferent condition across all trials (significant > indifferent: *F* = 29.87, *p *< 0.001, $\eta_{p}^{2}$ = 0.56).

**FIGURE 3:**
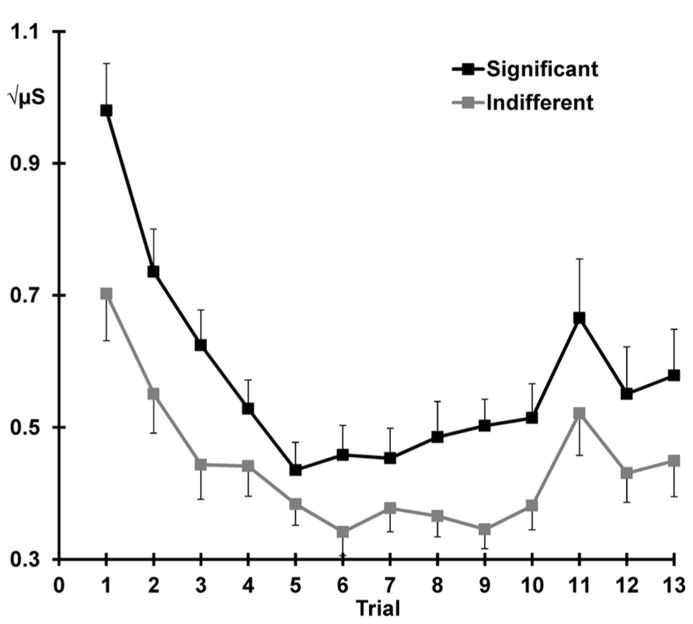
**Mean square-root transformed SCRs over trials for each condition, with standard error bars.** Decrement (trial 1–10), recovery (trial 11 > 10), and dishabituation (trial 12 > 10) of SCRs are apparent.

**Figure 3** illustrates the mean SCRs across all 13 trials separately for each condition. Across condition, response decrement was apparent with SCRs showing a linear decrease and plateau over trials 1–10 (linear trials: *F* = 91.60, *p *< 0.001, $\eta_{p}^{2}$ = 0.80; quadratic trials: *F* = 65.54, *p *< 0.001, $\eta_{p}^{2}$ = 0.74). The quadratic trend differed with condition (quadratic trials × significant > indifferent: *F* = 7.35, *p *= 0.012, $\eta_{p}^{2}$ = 0.24), suggesting that the indifferent responses continue to decline more systematically than the responses to the significant stimuli. Significant SCRs were also larger than SCRs to indifferent stimuli across the first 10 trials (significant > indifferent: *F* = 19.27, *p *< 0.001, $\eta_{p}^{2}$ = 0.46).

**Figure 3** also shows that response recovery was apparent, with larger SCRs for the deviant (trial 11) than the preceding trial 10 (10 < 11: *F* = 7.39, *p *= 0.012, $\eta_{p}^{2}$ = 0.24). SCRs were also larger over trials 10 and 11 for the significant than indifferent stimuli (significant > indifferent: *F* = 7.77 *p *= 0.010, $\eta_{p}^{2}$ = 0.25). There was no trial by condition interaction, indicating that the change in response from 10 to 11 did not differ with condition.

Dishabituation was evident, with larger SCRs at trial 12 compared to 10 (i.e., 10 < 12: *F* = 8.68, *p *= 0.007, $\eta_{p}^{2}$ = 0.27; **Figure 3**). Across trials 10 and 12, a main effect of condition was apparent with larger SCRs for the significant versus the indifferent condition (significant > indifferent: *F* = 8.29 *p *= 0.008, $\eta_{p}^{2}$ = 0.26). Again, there was no trial by condition interaction, indicating that dishabituation did not differ between the two conditions.

### SKIN CONDUCTANCE LEVELS

**FIGURE 4:**
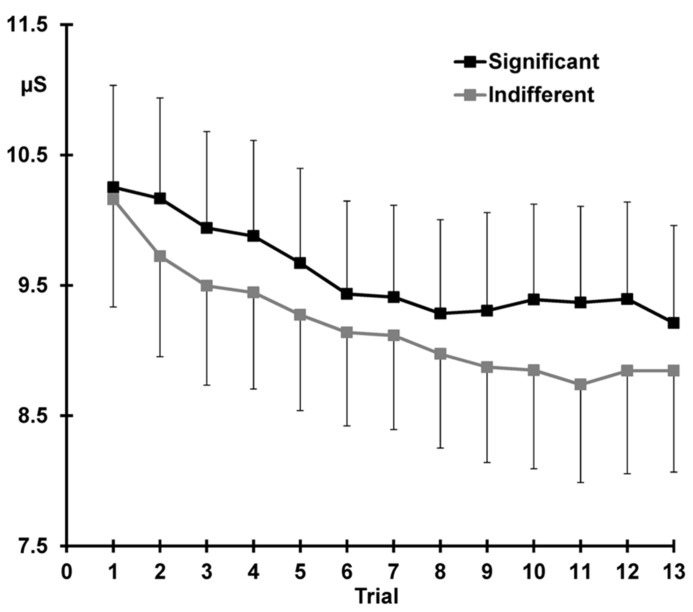
**Mean pre-stimulus SCL for each trial, again with standard error bars, separately for the two conditions.** Response decrement is apparent over the first 10 trials and sensitization can be seen after presentation of the deviant (trial 11) at trial 12.

**Figure 4** demonstrates that across the 13 trials, pre-stimulus SCL was greater for the significant than the indifferent condition (significant > indifferent: *F* = 10.06, *p *= 0.004, $\eta_{p}^{2}$ = 0.30).

Response decrement was evident, with SCLs over trials 1–10 demonstrating both linear and quadratic trends (linear trials: *F* = 41.50, *p *< 0.001, $\eta_{p}^{2}$ = 0.64; quadratic trials: *F* = 8.26, *p *= 0.009, $\eta_{p}^{2}$ = 0.26). There was a main effect of condition across trials 1–10, with larger SCLs for significant compared to indifferent stimuli (significant > indifferent: *F* = 6.70 *p *= 0.016, $\eta_{p}^{2}$ = 0.23). **Figure 4** shows that SCLs reached a plateau from around trial 8 in the significant condition, and around trial 11 in the indifferent condition, but these trends did not statistically differ with condition.

**Figure 4** shows that sensitization is apparent as an increase in SCL from trial 11–12 (11 < 12: *F* = 5.65, *p *= 0.026, $\eta_{p}^{2}$ = 0.20). SCLs were larger over both trials 11 and 12 for the significant than the indifferent condition (significant > indifferent: *F* = 9.25, *p *= 0.006, $\eta_{p}^{2}$ = 0.29). There was no trial by condition interaction.

### CORRELATIONS

**FIGURE 5:**
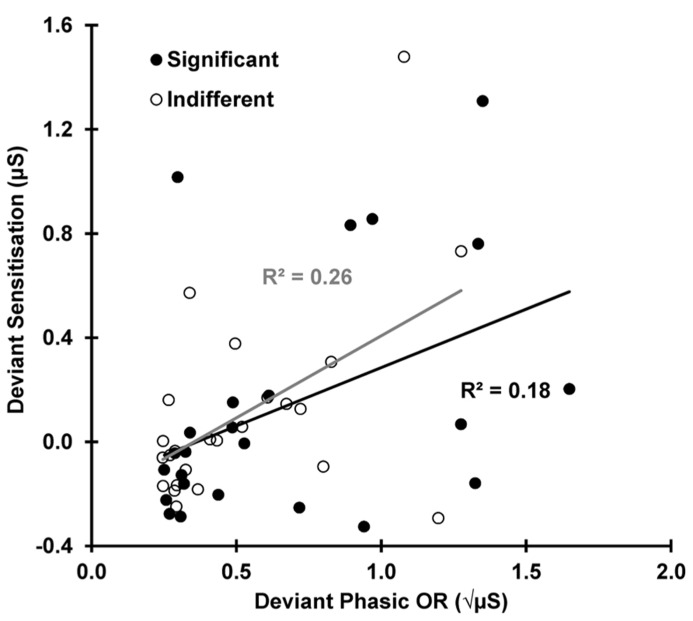
**Deviant sensitization (SCL: 12 minus 11) as a function of deviant ORs (SCR 11).** sensitization appears to be dependent on the deviant ORs for both conditions.

**FIGURE 6:**
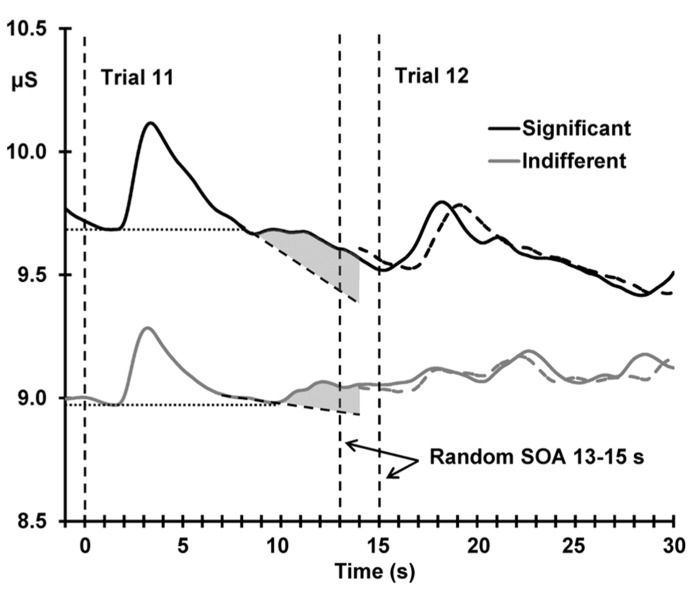
**Mean unbaselined electrodermal activity from 1 s preceding deviant stimulus onset, separately for the two conditions.** Vertical dashed lines represent stimulus onset; note the two dashed lines marking the variable onset of trial 12 due to the random SOA (13–15 s). The dashed waveforms represent the average unbaselined activity from the onset of trial 12; the slight shift in the latency of the response peak is directly related to the variable SOA. The horizontal dotted lines demonstrate the complete resolution of the SCR to the deviant stimulus for both conditions, and the diagonal dashed lines represent the continuation of this response. The shaded area illustrates the sensitization process.

**Figure 5** shows the deviant sensitization, the increase in SCL from trial 11–12, as a function of deviant OR. In both conditions, this SCL increase (deviant sensitization) is correlated with the deviant OR: indifferent *r*(22) = 0.510, *p* = 0.005; significant *r*(22) = 0.424, *p* = 0.019, suggesting a similar origin (the novelty associated with the deviant). However, **Figure 6** demonstrates that this sensitization is not just a remnant of the incomplete resolution of the phasic response to the deviant. **Figure 6** shows the average electrodermal activity across all subjects for trials 11 and 12 separately for each condition. The vertical dashed lines represent stimulus onset (including the variable SOA at trial 12). The horizontal dotted lines illustrate that the phasic SCR was complete and had returned to pre-response levels within 10 s from trial 11 onset, and the diagonal dashed lines represent the continuation of this response. The shaded area above this is sensitization. It can be seen that the dishabituated response at trial 12 is not directly affected by the previous OR at trial 11, as that response has already resolved. This indicates that any increase in arousal from trial 11–12 is sensitization rather than a remnant of the preceding phasic response.

**FIGURE 7:**
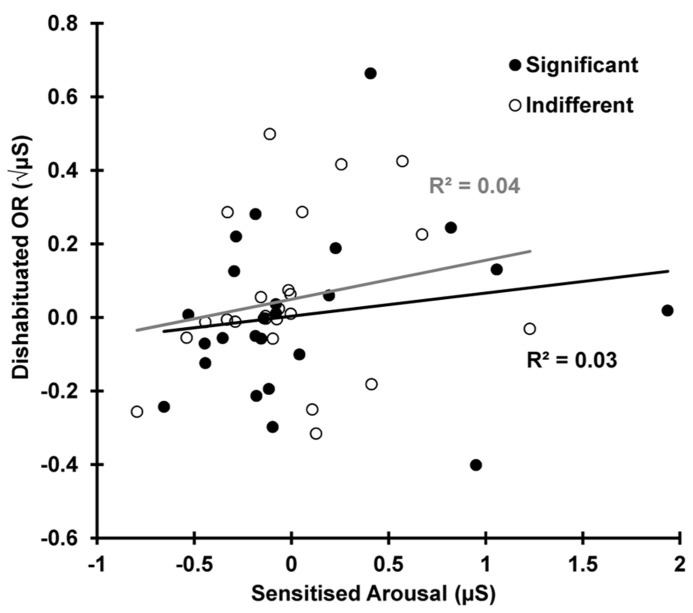
**Dishabituation as a function of sensitization for both conditions.** OR dishabituation is not due to the deviant-related sensitization.

Dishabituation is shown as a function of sensitization in **Figure 7**. This shows that the increase in SCR from trial 10–12 (dishabituation) is independent of the increase in arousal for the same two trials, 10–12 (sensitized arousal), for both the indifferent *r*(22) = 0.210, *p* = 0.162 and the significant condition *r*(22) = 0.173, *p* = 0.209.

**FIGURE 8:**
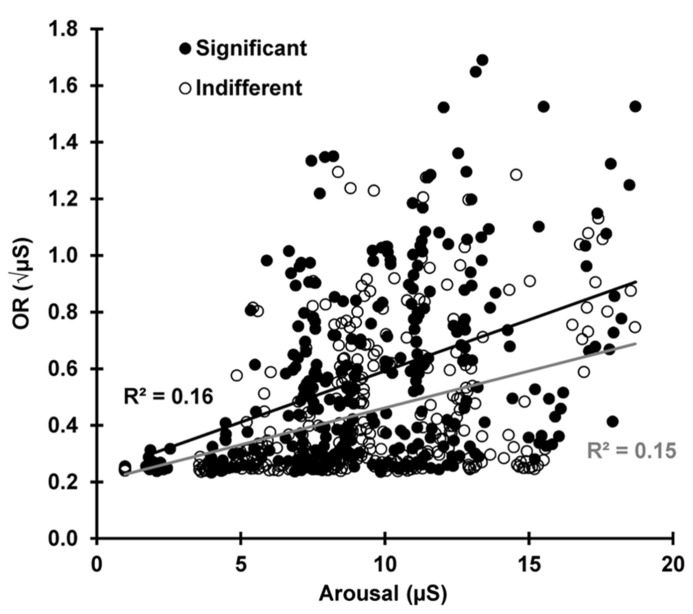
**For each subject and trial, the SCR is plotted against the pre-stimulus SCL, separately for indifferent and significant.** The OR is dependent on the current arousal level in both conditions.

To test the prediction that the current arousal level determines OR magnitude, **Figure 8** shows SCR plotted against pre-stimulus SCL for each subject and trial for both conditions (24 × 13 × 2 = 624 data points). Over all trials and subjects, SCR magnitude was dependent on the current SCL for indifferent *r*(310) = 0.383, *p* < 0.001 and significant *r*(310) = 0.406, *p* < 0.001 stimuli, indicating that the current arousal level amplifies the phasic OR. There was no statistical difference between the two conditions *z* = 0.340, *p* = 0.367.

**FIGURE 9:**
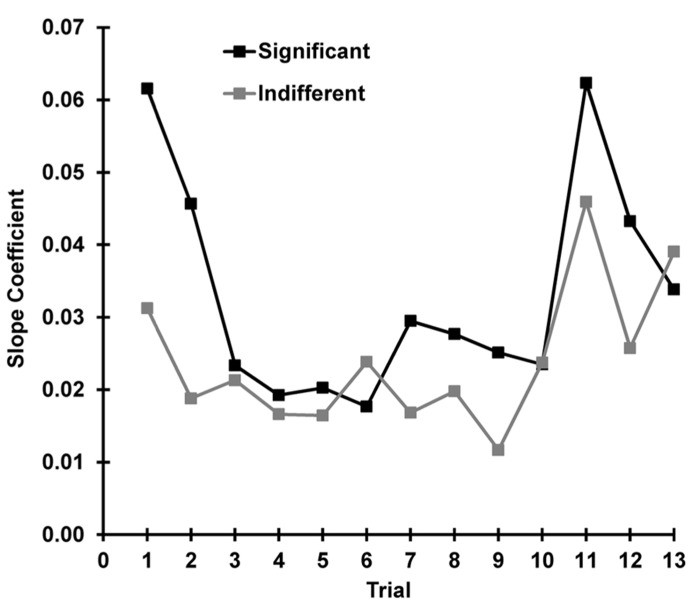
**For each trial, the SCR and pre-stimulus SCL were correlated.** The slope coefficients for this trial-by-trial comparison are plotted separately for each condition. The novelty and significance of the stimulus appears to determine the strength of the relationship between the OR and the current level of arousal.

To explore the relationship between state and response as a function of trial, separate correlations were carried out between SCR and pre-stimulus SCL for each trial (separately for indifferent and significant). These 13 correlations had a mean for indifferent stimuli of *r *= 0.403, *SD* = 0.13 and for significant of *r* = 0.434, *SD* = 0.16; these did not differ significantly *z* = -0.120, *p* = 0.452. **Figure 9** shows the slope coefficients of the underlying scatterplots for these correlations plotted over trials for each condition. The relationship between the OR and the current arousal level (i.e., the amplification factor) apparently changes with stimulus novelty and significance. This was tested with a multiple regression using our three hypothesized determinants of the SCR. Novelty, significance, and arousal accounted for 55% of the variance in SCR, and the linear combination of these three variables significantly predicted SCR, *F*(3, 620) = 90.38, *p* < 0.001. The coefficients for novelty (β = 0.302), significance (β = 0.125), and arousal (β = 0.030) were all found to significantly contribute to SCR [novelty: *t*(623) = 9.64, *p* < 0.001, significance, *t*(623) = 6.37, *p* < 0.001, arousal *t*(623) = 10.88, *p* < 0.001].

## DISCUSSION

The aim of this study was to determine the mechanism of dishabituation by testing separate predictions derived from dual process theory and Sokolov’s neuronal-model of the OR. Our previous investigation, Steiner and Barry (2011), utilized a dishabituation paradigm with a 5–7 s SOA, but this did not allow complete resolution of the phasic response preceding the dishabituation trial. In the current study, the extended SOA (13–15 s) allowed complete resolution of the response to the deviant trial, which served to demonstrate that sensitization, the change in arousal following the deviant, was genuine and not contaminated by the preceding deviant response. Dishabituation was not dependent on the change in arousal (sensitization) following the deviant, but instead, was dependent on the immediate pre-stimulus arousal level, as applies generally. This finding argues against dual process theory’s explanation of dishabituation and provides support for Sokolov’s assertion that dishabituation is a disruption to the habituation process.

As predicted, our phasic measure of the OR, the SCR, met the formal criteria for habituation (Thompson and Spencer, 1966; Rankin et al., 2009). That is, we observed *response decrement* over trials, *response recovery* to the interpolated deviant, and *dishabituation* to the representation of the habituated stimulus. The significance of the stimulus also affected OR magnitude, with larger SCRs for the significant compared to the indifferent condition. This is a robust finding that is consistent with previous research examining habituation of the electrodermal response (Barry, 2004, 2009; Steiner and Barry, 2011). Stimulus significance also affected the habituation of SCRs, with responses to indifferent stimuli showing a more systematic decline than SCRs to significant stimuli, a finding also in line with our previous investigations.

Our pre-stimulus state/arousal measure, SCL, decreased in magnitude over the first 10 trials and was greater over all trials for the significant compared to the indifferent condition, a finding consistent with Barry (2004). SCLs to significant stimuli did not continue to follow the same decremental pattern as the indifferent condition, and stayed somewhat elevated. It should be noted that the effects of stimulus significance (counting) observed here cannot be accounted for by Sokolovian or dual-process theories of the OR, as neither of these theories provide a mechanism for significance-related differences in tonic and phasic responding. Rather, an under-utilized theoretical concept, Maltzman’s voluntary OR, predicts that both arousal level and the OR will be enhanced for motivationally significant stimuli.

Sensitization of arousal was observed following the deviant, with larger pre-stimulus SCLs for the post-deviant trial compared to the deviant trial. This finding replicates Steiner and Barry (2011) and is in line with Barry (2004) and Barry and Sokolov (1993), where the sensitization process was explored at the start of a habituation sequence. Here, sensitization, measured as the change in arousal from trial 11–12, was positively correlated with the deviant phasic OR at trial 11. Initially, this seems to suggest that sensitization is dependent on the deviant OR in both conditions, but as shown in **Figure 6**, sensitization is independent of the preceding deviant trial. Steiner and Barry (2011) also found this positive correlation, but only for the significant condition; this was due to the incomplete resolution of the deviant OR. In that study, the outcome of this test was used to justify restriction of the exploration of the mechanism of dishabituation to indifferent stimuli only. However, in the current study, we extended the SOA beyond the time needed for the phasic response to resolve, and, as **Figure 6** shows, the OR to the deviant trial completely resolves in both conditions before the onset of the following stimulus. This suggests that sensitization is not directly dependent on the preceding OR to the deviant trial, but is rather an independent process occurring after this response has resolved.

Importantly, dishabituation was found to be independent of sensitization for both conditions, a finding extending Steiner and Barry (2011), where this was demonstrated for indifferent stimuli only. This argues against dual-process theory’s unique assertion that dishabituation is nothing more than a superimposed process of sensitization, independent of habituation. Rather, it suggests that dishabituation actually reflects the increased novelty associated with the reinstatement of the H-process post-deviant. This provides support for Sokolov’s assertion that dishabituation reflects a disruption to the habituation process.

We continued our investigation into the mechanism of dishabituation by exploring current arousal as Sokolov’s response-amplifier. We tested this by correlating the OR with the pre-stimulus arousal level for each trial and each subject for both conditions. The phasic OR was found to be dependent on the current state, and this did not differ with condition. This finding confirms the importance of arousal as a response amplifier and is consistent with findings from continuous performance tasks (VaezMousavi et al., 2007a,b). To examine how this relationship changed over trials, OR was correlated with current arousal at each trial for both conditions. When *r*-values were averaged across trials, there was no statistical difference between the two conditions. However, the slope of the correlation scatterplots (amplification) appeared to change as a function of trial, differing between indifferent and significant conditions. This seems to reflect the totality that current arousal, novelty, and significance together determine phasic response magnitude. This was confirmed with a multiple regression, where all three determinants of the OR were found to predict the magnitude of SCR.

In sum, our findings show that the novelty and significance of a stimulus, and the current level of arousal, consistently predict the magnitude of the phasic OR, including the dishabituated response. Here, novelty reflected dual process theory’s H-process, arousal was modeled on the S-process, and significance was based on Sokolov’s description of significant stimuli. Sokolov did not provide a theoretical mechanism for the significance effects observed here, so this was examined in the context of Maltzman’s voluntary OR. Importantly, we demonstrated that sensitization, the *change* in arousal, is a process that is separate from the resolution of the phasic response. Together, this suggests that dishabituation is a disruption of the habituation process, and the magnitude of this response is determined by the current arousal level.

The data presented here illustrate the process of response habituation and the influence of corresponding state changes in electrodermal activity. When examining slow-changing autonomic measures, such as the electrodermal response, the SOA should be long enough to ensure the complete resolution of responses. To further disentangle the phasic and tonic components of the OR, work integrating slower autonomic measures (e.g., electrodermal activity) with faster-changing central (e.g., EEG) measures of habituation and arousal is required. For example, using a dishabituation task, Barry et al. (2012) showed that an initial stimulus-induced transient increase in delta and theta EEG activity correlated with SCR, showing response decrement, recovery, and dishabituation. Future research should seek to confirm the central-neural mechanism of dishabituation. In that case, dishabituation of central measures, such as components in the event-related potential of the brain, should reflect a change in cortical excitation, demonstrable with EEG measures. Current work in our laboratory is exploring this.

## Conflict of Interest Statement

The authors declare that the research was conducted in the absence of any commercial or financial relationships that could be construed as a potential conflict of interest.
